# Understanding Gender Differences in Acceptance of Intimate Partner Violence Against Women: Are Women Truly More Accepting Than Men?

**DOI:** 10.1177/08862605251324964

**Published:** 2025-03-14

**Authors:** Joseph A. Kilgallen, Susan B. Schaffnit, Yusufu Kumogola, Mark Urassa, David W. Lawson

**Affiliations:** 1 Department of Anthropology, University of California, Santa Barbara, USA; 2 National Institute for Medical Research, Mwanza, Tanzania

**Keywords:** intimate partner violence/abuse, attitudes/perceptions, research methodology/measurement, gender-based violence

## Abstract

A surprisingly consistent finding from the global health literature on physical intimate partner violence against women (IPVAW) indicates that women self-report greater agreement with statements justifying IPVAW than men. This pattern has been interpreted as evidence of women’s internalization of inequitable gender norms and used to support the development of intervention programs that target harmful beliefs about the acceptability of IPVAW among men and women. Here, we propose an alternative explanation that apparent gender differences in the acceptance of IPVAW reflect an artifact of social desirability bias. To investigate this proposition, we utilize attitudinal data on IPVAW from 317 married couples in Northwestern, Tanzania, combining a conventional self-report measure and a novel indirect wife-reported measure of men’s attitudes, which we suggest represent a more accurate representation of men’s true beliefs. Consistent with prior studies, comparisons of self-reported beliefs indicate that women report greater acceptance of IPVAW than men. However, the direction of this difference is reversed when men’s beliefs are measured using indirect wife-reported estimates, with men accepting IPVAW more than women. Our results provide a parsimonious explanation to a widely reported, but paradoxical, finding in the IPVAW literature, and highlight the inadequacies of self-report data in research on sensitive topics. We suggest that future studies of IPVAW more strongly weigh the relevance of social desirability bias and invest in the continued development of indirect and mixed-methods designs in the measure of IPVAW attitudes and behavior.

## Introduction

This short report re-examines a common, but paradoxical, finding from the global health literature on physical intimate partner violence against women (hereafter “IPVAW”). Women routinely self-report greater agreement with statements expressing justification of IPVAW than men, particularly in low- and middle-income country (LMIC) settings where acceptance of IPVAW is at its highest among both men and women (Alabi & Ramsden, 2021; Koenig et al., 2003; Krause et al., 2016; Paintsil et al.,, 2023; Sandberg et al., 2020; Sardinha & Catalán, 2018; Tran et al., 2016; Uthman et al., 2009; Waltermaurer, 2012; Yount et al., 2014). Throughout we refer to this phenomenon as a higher acceptance of IPVAW among women, recognizing that agreement with such statements need not necessarily imply a blanket condonement or justification of IPVAW, or that it is a desirable behavior, but rather only that it is considered acceptable in specific scenarios. At face value, a higher acceptance of IPVAW among women is puzzling since women suffer an undeniable cost from IPVAW, and from patriarchal norms in general. Explanations for observed gender differences in acceptance of IPVAW are typically grounded in concepts of gender socialization (Stockard, 2006) and patriarchal bargaining (Kandiyoti, 1988). From this perspective, women adopt or “internalize” prevailing gender norms that ostensibly contradict their own interests, particularly in settings where women are positioned as economic dependents and men as enforcers of obedience, such that challenging male authority is risky while compliance offers relative benefits (Eckenrode, 2018; Sardinha & Catalán, 2018; Sunmola et al., 2020; Yount et al., 2013, 2014). Furthermore, if exposure to alternative cultural norms or “scripts” that disapprove of IPVAW is increased by factors such as globalization and urbanization (Charles, 2019), and men are more subject to these influences via higher rates of education and labor market participation, these increased rates of exposure could account for women’s overall greater acceptance of IPVAW in some contexts (Tran et al., 2016).

Here, we propose an alternative explanation that apparent gender differences in the acceptance of IPVAW reflect measurement error due to social desirability bias, that is, the tendency to respond to survey questions in ways deemed favorable by researchers (Krumpal, 2013). Social desirability bias can be divided into two often-associated phenomena which may differ in relevance for alternative contexts but are not always easy to distinguish analytically (Visschers et al., 2017). First, *self-deception* refers to the unconscious inclination to view oneself favorably, this is characterized by a sincere, positive bias where individuals genuinely believe their reports to safeguard their self-esteem and self-image (Dutton & Hemphill, 1992). Second, *impression management* is the purposeful presentation of a false persona, for example, intentionally altering one’s responses in an interview to project a more favorable image (Zerbe & Paulhus, 1987). Social desirability bias, particularly in the form of impression management, is expected to be strongest in circumstances that are (i) not anonymous, such as in conventional face-to-face surveys which are the norm for IPVAW surveys in LMIC settings (e.g., in the Demographic and Health Surveys); (ii) when responses risk implicating oneself as a performer of an undesirable behavior; and (iii) when participants are aware of a contradiction between their own beliefs and those deemed desirable by others. As such, with men being the main perpetrators of IPVAW, and in some cases also more aware than women of national laws prohibiting IPVAW (Karim et al., 2023; Yount et al., 2014), ostensibly lower acceptance of IPVAW among men than women in self-report surveys may be explained as an artefact of social desirability bias.

Consistent with our hypothesis, IPVAW research has long highlighted the unreliability of self-report data on IPVAW attitudes and behavior in both men and women (Abramsky et al., 2022; Gibson et al., 2022; Halim et al., 2018; Singh et al., 2022). For example, Singh et al. (2022) report strong interviewer effects on acceptance of IPVAW in India, suggesting that small differences in how researchers interact with participants influence responses. Abramsky et al. (2022) document that a large proportion of sampled women provided discrepant reports about their own past experiences of IPVAW in a Tanzanian longitudinal study. Also in Tanzania, Halim et al. (2018) report that around a third of couples disagreed about past occurrence of IPVAW (see also Kilgallen et al., 2022), and Mchome et al. (2024) further highlight how social desirability bias impacts qualitative research; with men participating in discussions on IPVAW acknowledging that it is widespread, but never personally admitting to committing it themselves. However, to our knowledge, no study has explicitly considered the role of social desirability bias in accounting for observed gender differences in IPVAW attitudes. To do so requires contrasting measurement tools that differ in their vulnerability to social desirability bias. If our hypothesis is correct, gender differences observed in self-report data should attenuate or reverse when using alternative methods more likely to reveal honest responses. In this vein, a recent study by Gibson et al. (2022) used the list randomization technique, an indirect measure used to increase respondent privacy, to demonstrate “concealed acceptance” of IPVAW in Ethiopia. Conventional self-report measures showed the typical pattern of higher acceptance of IPVAW among women (*20% of women and 15% of men accepted IPVAW*), but their indirect method shows both higher overall IPV acceptance and a reversed gender difference (*26% of women and 32% of men accepted IPVAW*). However, neither difference was reported to be statistically significant, and the list randomization technique is vulnerable to Type II errors unless samples are very large, making it difficult to draw firm conclusions from this result.

Here, we apply a novel indirect approach to addressing social desirability bias that does not require very large samples, that is, asking men’s wives to confidentially report on their husband’s beliefs about IPVAW (Lawson et al., 2021). Applying this approach to a semiurban Tanzanian community, we predict that conventional self-report measures will show higher acceptance of IPVAW among women than men, while our indirect method will demonstrate the reverse pattern with higher acceptance of IPVAW among men. While our study draws on a modest sample size and data from only a single community, this methodological innovation provides a rare opportunity to investigate the role of social desirability bias in accounting for gender differences in the acceptance of IPVAW.

## Methods

### Study Context

All data were collected in June to August 2019 within a single semiurban community within the boundaries of the Magu Health and Demographic Surveillance System (HDSS), situated in northwestern Tanzania, approximately 20 km east of Mwanza city. The HDSS has monitored the population of over 35,000 residents across several villages since 1994 (Kishamawe et al., 2015; Urassa et al., 2024). Most of the residents belong to the Sukuma ethnic group (Wijsen & Tanner, 2002), although other ethnic groups have moved to the area due to the influence of urbanization and globalization. Traditionally, the Sukuma were agropastoralists, and the practice continues today, but men and women are increasingly engaging in different livelihood activities, working as skilled laborers, petty traders, or in small businesses (entrepreneurs; Lawson et al., 2021). Sukuma customs can be characterized as traditionally patriarchal (Wijsen & Tanner, 2002), with adolescent boys generally enjoying more support and favor than girls, and an expectation that adult men are the primary breadwinners, holding more power and decision-making authority than women. Women and adolescent girls are traditionally expected to be engaged in household work such as cooking, taking care of children, and other domestic chores. However, beliefs and behaviors regarding gender roles are changing, particularly in more urban areas, and are accompanied by a rise in both female education and labor market participation (Ishungisa et al., 2024; Kilgallen et al., 2022; Lawson et al., 2021). Previous research suggests that IPVAW in the wider Mwanza region is commonplace and, under many circumstances, deemed socially acceptable. For example, one recent study carried out in the Mwanza region found that around 60% of women report experiencing some form of IPVAW in their lifetime (Abramsky et al., 2019), and past research in the study community established that approximately 38% of women self-report having experienced IPVAW within the last 12 months (Kilgallen et al., 2022).

### Sampling

The 2018 Magu HDSS census (Urassa et al., 2024) served as a sampling frame, providing a systematic method for identifying individuals who met our inclusion criteria: married couples with at least one child, where the man was projected to be between the ages of 25 and 40. This limited age range was chosen to maximize our ability to explore variation in attitudes independently of potential age/cohort effects, and the criteria of having at least one child reflected wider study aims to investigate how gender role ideology relates to family structure (see Lawson et al., 2021). The Magu HDSS, established in 1994, conducts longitudinal demographic surveillance of all households in seven contiguous villages within one administrative ward in Magu district, Mwanza region. The HDSS follows a standardized protocol, collecting household-level demographic data through home visits every 8 months, recording births, deaths, and migration patterns, making it a reliable and structured sampling frame. Working from the largest, more urban, community within the HDSS, we identified 1,275 eligible husband–wife pairs and initially aimed for a random sample of 400. Working through our sampling frame, we visited each couple’s residence and, if present interviewed on location. If sampled individuals were absent, present family or neighboring community members were asked about their whereabouts (e.g., a place of work), and if within the immediate range of the study community we made effort to find them. Sampling in this way proved challenging given frequent daily movements and longer-term migrations, particularly of men who were often away from home in the daytime. Consequently, our sample size fell short of our target at 317 husband–wife pairs and is best considered a convenience rather than fully random sample, biased toward men found at home or within close range. In rare cases of polygynous marriage, one wife was chosen for interview, based on immediate availability. Occasionally, men provided ages that did not match the HDSS. If the man’s self-reported age at the time of survey was within 5 years of our selection criteria, he was included in our final sample (i.e., men aged 20–45 years were included).

### Surveys

Surveys were completed on tablets using Open Data Kit (Hartung et al., 2010). Before conducting any research activities, participants were provided with a copy of the informed consent document which was read aloud to the participant by the researcher. Researchers emphasized that participation was voluntary and gave the participant ample time to ask any questions before collecting written consent. Upon written consent, all participants were interviewed in private settings by Tanzanian researchers of the same gender, in the local language of Kiswahili. Participants first provided basic sociodemographic data and then were asked about their attitudes about IPVAW as part of a longer attitudinal survey assessing multiple dimensions of gender role ideology (Lawson et al. 2021). To measure support for IPVAW, men and their wives were then read three statements (1. “*A man is justified in hitting his wife if she argues with him*”; 2. “*A man is justified in hitting his wife if she refuses to have sex with him*”; and 3. “*A woman should tolerate being beaten by her husband to keep her family together*”) and asked to state if they strongly agree, agree, neither agree nor disagree, disagree, or strongly disagree with each. When a participant stated that they agreed/disagreed, they were asked to clarify whether this was a mild or strong level of agreement/disagreement. Participants were informed that some of the questions might be about sensitive topics, and that they were free to skip questions if uncomfortable. Field assistants were also trained to look out for signs that a participant might be uncomfortable and to ask the participant if they would prefer to skip the question if so. After reporting their own beliefs, men’s wives were asked to report if they felt their husband would agree/disagree with each of the three statements using the same Likert scale.

Ethical approval for this study was granted by University of California, Santa Barbara (UCSB)’s Office of Research (4-19-0247), the Tanzanian National Institute for Medical Research Lake Zone Institutional Review Board (MR/53/100/595), and the Tanzanian National Ethical Review Committee (NIMR/HQ/R.8a/Vol.IX/3104). Community-level approval was also sourced by a presentation of study objectives to village leadership. Analysis was conducted in R (R Core Team, 2020), using Wilcoxon rank-sum tests to compare men’s and women’s acceptance of IPVAW. Likert scale data are inherently ordinal, meaning the intervals between response options cannot be assumed to be equal (Jamieson, 2004), thus the Wilcoxon rank-sum test was chosen as it is robust for analyzing ordinal data and does not rely on assumptions of normality or equal variance (Allen & Seaman, 2007; Conover, 1999).

## Results

We collected data on 317 husband–wife pairs. The mean age of men was 35 years, with wives typically several years younger than their husbands. Husband–wife pairs reported roughly equivalent levels of absolute educational attainment, with most completing only primary school (nearly 70%), and around 20% completing secondary school. Considering the relative length of education reveals greater discrepancies, close to two-thirds of couples (61%) reported that the husband had more years of education, 11% of couples reported equal years of education, while the wife had more years of education in one in four marriages (27%). Fertility was variable but overall high, with 43% of couples having four or more children. For further descriptive data on this sample see Kilgallen et al. (2022). For our IPVAW questions, response rates were high with only 1% to 2% of participants stating they did not know or refusing to declare their own level of agreement with each statement. Refusal rates were also low when asking women to estimate their husband’s agreement with each statement; however, between 4% and 7% stated that they did not know their husbands’ beliefs, depending on the statement. These cases were removed from further analysis.

**Figure 1.**
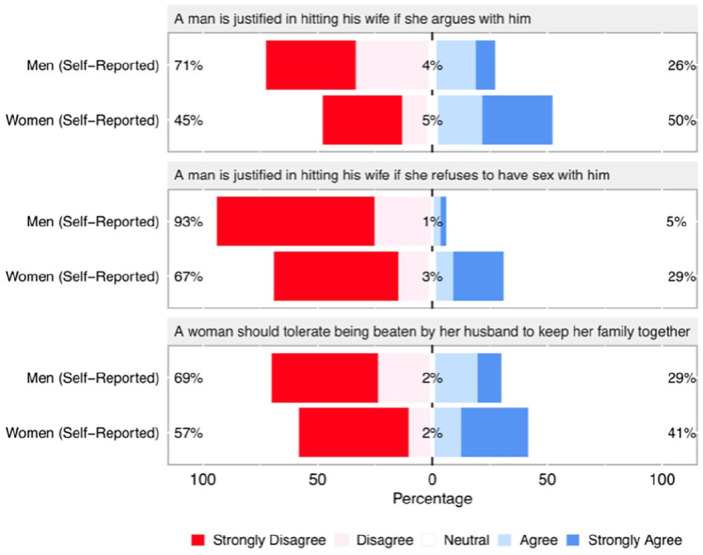
Men’s self-reported and women’s self-reported agreement with statements justifying IPVAW. According to conventional self-report measures, women are more likely than men to agree with all three statements supporting IPVAW (see text for statistical test results).

Consistent with past research, we find that men’s self-reported agreement was lower compared to women’s self-reported agreement across all three statements describing IPVAW (Figure 1). A Wilcoxon test shows that apparent gender differences are statistically significant across all three statements (“*A man is justified in hitting his wife if she argues with him: W* = 38,832, *p* < .0001, *n* = 632; “*A man is justified in hitting his wife if she refuses to have sex with him*”: *W* = 37,456, *p* < .0001, *n* = 627; “*A woman should tolerate being beaten by her husband to keep her family together*”: *W* = 45,782, *p* < .05, *n* = 633). Apparent differences are striking in magnitude with, for example, 50% and 29% of women agreeing that a man is justified hitting his wife if she argues or refuses to have sex with him, respectively, compared to just 26% and 5% of men self-reporting agreement with the same statements.

**Figure 2.**
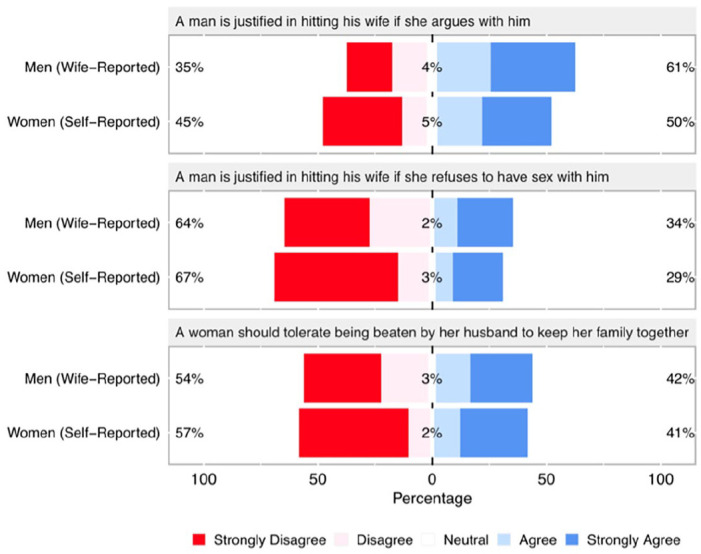
Men’s wife-reported and women’s self-reported agreement with statements justifying IPVAW. According to our indirect wife-reported measure of men’s beliefs women are less likely to agree with the first two statements supporting IPVAW, while agreement with the third statement does not differ by gender (see text for statistical test results).

Substituting our original self-report measure of men’s agreement with statements justifying IPVAW with our indirect wife-reported measure presents a very different picture (Figure 2). A Wilcoxon rank sum test shows that, according to men’s wives, men were more likely than women to agree in two out of the three statements justifying IPVAW (“*A man is justified in hitting his wife if she argues with him*”: *W* = 53,800, *p* < .001, *n* = 614; “*A man is justified in hitting his wife if she refuses to have sex with him*”: *W* = 53,111, *p* < .001, *n* = 612), while the third statement shows no gender difference (“*A woman should tolerate being beaten by her husband to keep her family together*”: *W* = 49,304, *p* = .09, *n* = 606). Differences are relatively small in magnitude, but still meaningful; according to women, for example, 57% and 33% of their husbands agree that a man is justified hitting his wife if she argues or refuses to have sex with him, respectively, compared to 50% and 29% of women self-reporting agreement with these same statements.

## Discussion

Decades of research on IPVAW has found that women routinely self-report greater agreement with statements expressing justification of IPVAW than men, particularly in LMIC settings where acceptance of IPVAW is at its highest among both men and women (Alabi & Ramsden, 2021; Koenig et al., 2003; Krause et al., 2016; Paintsil et al.,, 2023; Sandberg et al., 2020; Sardinha & Catalán, 2018; Tran et al., 2016; Uthman et al., 2009; Waltermaurer, 2012; Yount et al., 2014). We have hypothesized that this phenomenon is an artifact of measurement error due to social desirability bias. Consistent with our hypothesis, using an indirect survey method to assess men’s attitudes reverses observed patterns, with men showing greater agreement with statements justifying IPVAW than women. Similar, although not statistically significant patterns, have been recently revealed in a study of IPVAW attitudes in Ethiopia (Gibson et al., 2022). As part of a larger study of gender role ideology at the same study site, Lawson et al. (2021) have also demonstrated a wider tendency for men to self-report greater agreement with a broader range of statements supporting women’s empowerment than is estimated indirectly by wife-reported measures. Furthermore, in related qualitative work, Ishungisa et al. (2024) found that male focus group participants admit to strategically misrepresenting their own beliefs and behaviors relating to gender roles to appear favorable to others. It therefore seems feasible that social desirability bias in men’s responses, particularly in the form of impression management, is pervasive and may account for why past studies have found that women not only self-report greater acceptance than men for IPVAW but also wider aspects of gender inequality (Palermo et al., 2020).

Beyond gender differences, our findings also reveal notable variation in the acceptance of IPVAW depending on the justification provided. Consistent with previous work conducted across sub-Saharan Africa and Vietnam (Alabi & Ramsden, 2021; Krause et al., 2016; Paintsil et al., 2023), we found greater acceptance of statements justifying violence when a woman argues with her husband than when she refuses sex. This difference may stem from the perception that arguing represents a direct challenge to male authority, whereas refusing sex may be seen as excusable under certain circumstances. In fact, previous research conducted in Tanzania highlights that gender norms often justify a husband physically disciplining his wife to assert his authority as the head of the household, and to reinforce the expectation that women should be submissive to their partners (Jakobsen, 2015). In contrast, a wife’s refusal to have sex may be viewed as a less acceptable reason for physical violence, as some of our participants anecdotally noted that a wife may have legitimate reasons for refusing sex such as illness or exhaustion. However, qualitative research in Tanzania also suggests that sexual IPVAW is frequently condoned by gender norms emphasizing men’s entitlement to sex from their partners (McCleary-Sills et al., 2016). These findings highlight the nuanced and context-specific nature of IPVAW attitudes, suggesting that acceptance may vary significantly depending on the type of violence.

Our study is not without limitations. We draw on a modest sample size and data from only a single community which may limit the generalizability of our findings. Additionally, due to difficulties in recruitment, our sample is biased toward individuals closer to their homes who were easier to locate and thus is not fully representative. Our indirect “wife-reported” method of assessing men’s attitudes also has its’ shortcomings. We have assumed that women have a lower incentive to misrepresent their own beliefs than men, since they are not perpetrators of IPVAW and may also be less aware that declared acceptance of IPVAW would contradict the views of the research team (Karim et al., 2023; Yount et al., 2014). However, we caution that women may also have reason to misrepresent their husbands’ views, or simply be uninformed or unaware of his beliefs; after all, 4% to 7% of women reported that they did not know what their husband believed depending on the survey question. It is possible, for example, that women avoid stating their husband supports IPVAW to sidestep a perceived risk of being reprimanded for reporting negatively on their spouse. Yet, such a bias would only lead us to further underestimate the true extent to which men agree with statements justifying IPVAW, and so cannot account for the reversed gender difference we report. We suggest that the most parsimonious explanation of our observed results is that, contrary to numerous studies carried out across many LMIC contexts (Alabi & Ramsden, 2021; Koenig et al., 2003; Krause et al., 2016; Paintsil et al.,, 2023; Sandberg et al., 2020; Sardinha & Catalán, 2018; Tran et al., 2016; Uthman et al., 2009; Waltermaurer, 2012; Yount et al., 2014), men in fact agree with statements justifying IPVAW more than or at least as much as women. This conclusion is consistent with IPVAW being directly costly to women and a part of a wider patriarchal ideology that represents a conflict of interest between the genders (Lawson et al., 2023).

Future studies should continue to weigh the relevance of social desirability bias and invest in the continued development of indirect and mixed-methods designs in the measure of IPVAW attitudes and behavior, and sensitive topics more generally (see also Abramsky et al., 2022; Gibson et al., 2022; Halim et al., 2018; Singh et al., 2022). The list randomization technique recently employed by Gibson et al. (2022) to assess attitudes to both IPVAW and female genital cutting/mutilation (Gibson et al., 2018) may be particularly fruitful where the required large sample sizes are possible (see also Castilla & Murphy, 2022). Often approaches such as the use of implicit association tests may also be useful (Nillesen et al. 2021; Sanchez-Prada et al., 2021); however, complex survey tools can also be difficult for researchers to implement and participants to comprehend (see Hruschka et al., 2018 for a relevant discussion of mismatch between survey instruments and cultural differences in skills, motivations, and modes of social interaction in field research). Our approach, while not without its own shortcomings, has the advantage of being relatively simple to implement and understand as well as not requiring especially large sample sizes. Ultimately, we argue that to gain the greatest confidence in interpreting survey data, an investment in mixed-methods designs that also facilitate in-depth qualitative investigation of men and women’s attitudes is essential.

## Conclusion

We present evidence that the common observation that women declare greater agreement with statements justifying IPVAW than men is an artefact of social desirability bias. If our interpretation is correct, this resolves an enduring but paradoxical finding in the global health literature on IPVAW and underscores a general requirement for caution when interpreting self-report data on sensitive topics more generally. Assuming our findings are generalizable, from a programmatic perspective we conclude that initiatives attempting to shift harmful beliefs about IPVAW should continue to primarily focus on men, rather than targeting women as some have suggested (Paintsil et al., 2023). Women may often agree with statements that justify IPVAW, and the concepts of norm internalization and patriarchal bargaining likely help to explain their beliefs, but we suggest applying skepticism to reports emphasizing that women accept IPVAW more than men. Furthermore, social norm interventions that aim to “correct” misperceived perceptions about men’s apparent low acceptance of IPVAW or other related aspects of gender inequality, should be cautious of the potential to misinform community members. Lawson et al. (2024), for example, have recently shown that a tendency for men to overestimate how many of their male peers accept IPVAW is apparent when using self-reported beliefs to measure prevailing norms, but absent when using wife-reported estimates of men’s beliefs (see also Ishungisa et al., 2024). We conclude by encouraging further investment in mixed-methods designs and the development of indirect measurement techniques in the study of IPVAW and monitoring and evaluation of related initiatives and programs (Cloward, 2014; Gibson et al., 2018, 2022; Lawson et al., 2021; Lindstrom et al., 2010; Nillesen et al., 2021).
